# Food protein-induced enterocolitis syndrome (FPIES) following COVID-19 infection: a case report

**DOI:** 10.3389/falgy.2025.1705278

**Published:** 2025-11-17

**Authors:** Fayhan AlRoqi, Mohammed S. AlSanad, Abdulrahman N. AlJaber

**Affiliations:** 1 Division of Pediatric Allergy and Immunology, Department of Pediatrics, King Abdullah Specialized Children’s Hospital, Riyadh, Saudi Arabia; 2 King Abdullah International Medical Research Center, Ministry of National Guard-Health Affairs, Riyadh, Saudi Arabia; 3 College of Medicine, King Saud bin Abdulaziz University for Health Sciences, Riyadh, Saudi Arabia

**Keywords:** FPIES, COVID-19, amino acid–based formula, oral food challenge, food allergy

## Abstract

**Introduction:**

Food protein-induced enterocolitis syndrome (FPIES) is a non-IgE-mediated form of food allergy characterized by gastrointestinal manifestations following ingestion of the offending food. Most cases are identified during the first year of life, most frequently triggered by cow's milk or soy; however, alternative clinical phenotypes beyond this classic presentation have also been reported. In this case, we report a patient who developed acute FPIES to cow's milk ingestion following a COVID-19 infection, despite previous tolerance to cow's milk. This case raises the hypothesis that viral infections such as COVID-19 may act as cofactors or unmasking events in the development of FPIES.

**Case presentation:**

We report a 10-month-old boy who experienced recurrent episodes of profuse vomiting, followed by persistent diarrhea, beginning at 25 days of age—just a few days after a COVID-19 viral illness—with subsequent resolution upon transition to an amino acid–based formula. An oral food challenge (OFC) with cow's milk triggered repetitive emesis within 2 h of ingestion, accompanied by pallor, lethargy, severe diarrhea, and hypotension, which required multiple fluid boluses. The patient was admitted to the intensive care unit for monitoring of FPIES complicated by fluid-responsive hypovolemic shock. Clinical improvement was observed within 24 h of re-initiating amino acid–based formula, and the patient was discharged after 48–72 h with complete resolution of symptoms.

**Conclusions:**

A review of the literature revealed no prior reports of FPIES precipitated by viral infections. This case highlights a noteworthy temporal association between COVID-19 infection and the subsequent onset of FPIES in a patient who had previously tolerated cow's milk formula. Further studies are warranted to explore the possibility of viral infection induced FPIES.

## Introduction

Food protein–induced enterocolitis syndrome (FPIES) is a non–IgE-mediated food allergy characterized by delayed onset of symptoms. The hallmark manifestation is vomiting occurring 1–4 h after ingestion of the offending food. Acute episodes may also present with lethargy, pallor, and diarrhea. Diagnosis can be challenging, as the delayed onset of the symptoms often obscures the association with the triggering food (1).

FPIES typically presents during the first year of life, with the most frequently reported triggers in infants being cow's milk, soy, rice, and oat, followed by various fruits, vegetables, and eggs. In adults, seafood is the most common eliciting food. Symptoms usually appear with the introduction of formula or solid foods, predominantly affecting formula-fed infants and young children. Fewer than 5% of cases have been described in exclusively breastfed infants, as reported by Mehr et al. (2).

FPIES was first described by Powell in 1976 as a delayed-onset enterocolitis in two infants who developed recurrent vomiting, bloody diarrhea, abdominal distension, septic-like appearance, and hypothermia following ingestion of cow's milk and soy-based formulas (1). The diagnosis of FPIES is primarily clinical, based on a history of characteristic symptoms and resolution upon elimination of the suspected trigger food. Other potential causes must be excluded, and oral food challenges (OFCs) remain the gold standard for diagnosis, particularly in cases with ambiguous clinical histories.

In this case, we report a patient who developed acute FPIES to cow's milk ingestion following a COVID-19 infection, despite previous tolerance to cow's milk. This case highlights the need for early recognition and intervention when evaluating patients with post-infectious gastrointestinal manifestations.

## Case presentation

The patient is a 10-month-old male infant with Down syndrome, known to have a small ventricular septal defect (VSD) and an atrial septal defect (ASD), neither of which required medical intervention. He was born full-term via spontaneous vaginal delivery without complications and was discharged 2 days later after confirmation of Down syndrome by FISH analysis. Since birth, he had been feeding well on a combination of breast milk and standard cow's milk formula without complications. He had demonstrated normal growth and feeding tolerance without any gastrointestinal symptoms. No changes in formula brand, composition, or feeding frequency occurred prior to the onset of symptoms.

At 20 days of age, he was admitted with fever, upper respiratory tract infection, and diarrhea for evaluation to rule out sepsis. A full sepsis workup was performed, revealing COVID-19 infection. He was hospitalized for 2 days and discharged in stable condition after negative culture results. Four days later, he returned with persistent vomiting and worsening diarrhea. Examination was unremarkable except for signs of moderate-to-severe dehydration. He was admitted to the pediatric intensive care unit (PICU) with hypovolemic shock, metabolic acidosis, hypoglycemia, and elevated methemoglobin levels. He remained hospitalized for 11 days with a working diagnosis of viral acute gastroenteritis and possible lactose intolerance. Initial management with a lactose-free formula was ineffective, but symptoms improved following transition to an amino acid-based formula (AAF). He remained on AAF with resolution of vomiting and diarrhea, achieving appropriate weight gain and normal developmental milestones. At 10 months of age, he was referred to the Allergy Clinic for further evaluation.

The initial impression was milk intolerance secondary to viral gastroenteritis with suspected lactose intolerance. His history was inconclusive for IgE-mediated reactions, and there was no clear indication for initiating an amino acid–based formula at that time as he was tolerating regular formula prior to COVID-19 with no concerns. Given the clinical context, an open oral food challenge (OFC) was performed to evaluate for a non–IgE-mediated reaction such as FPIES. The patient received 90 mL of cow's milk formula, and after 95 min developed repetitive vomiting (five episodes), accompanied by pallor and four episodes of large-volume diarrhea. He subsequently developed moderate-to-severe dehydration and hypotension, requiring intravenous fluid boluses of 60 mL/kg. He was admitted to the PICU for ongoing monitoring, hydration, and standby inotropic support.

A baseline CBC was obtained to monitor neutrophil counts. Neutrophils increased from 2.4 × 10^3^ /μl at baseline to 8.4 × 10^3^ /μl after 8 h, with no significant rise in methemoglobin levels. The following day, he was transferred to the ward after 24 h of NPO status and was restarted on AAF, resulting in complete resolution of vomiting and diarrhea.

A diagnosis of cow's milk triggered FPIES was confirmed through an oral food challenge, with complete clinical improvement following elimination of the offending agent. The diagnosis of acute FPIES was established based on the international consensus criteria described by Nowak-Wegrzyn et al. (3). The major criterion**—**repetitive vomiting occurring 1–4 h after ingestion of the suspected food in the absence of IgE-mediated symptoms—was fulfilled, as the patient developed multiple episodes of vomiting after cow's milk ingestion without urticaria, angioedema, or respiratory distress. Several minor criteria were also met, including lethargy, pallor, hypotension requiring intravenous fluid boluses, and diarrhea within 24 h of ingestion. Complete resolution of symptoms following elimination of cow's milk further confirmed the diagnosis. Stool polymerase chain reaction (PCR) testing during both the COVID-19 infection and the subsequent FPIES episode was negative. The family was advised to continue amino acid–based formula and to plan a supervised rechallenge after 1–2 years, as the majority of patients outgrow FPIES by this age.

## Discussion

Although first described in the 1970s, food protein–induced enterocolitis syndrome (FPIES) remains poorly understood and is likely underdiagnosed (4). FPIES is a non–IgE-mediated gastrointestinal food hypersensitivity disorder. In response to specific food proteins, intestinal lymphocytes release inflammatory cytokines, leading to increased intestinal permeability, malabsorption, dysmotility, vomiting, diarrhea, abdominal pain, and potential failure to thrive (5). The reported incidence of FPIES ranges from 0.015% to 0.7% (6–8).

The disease typically presents in two forms. Almost all patients experience vomiting, which is typically projectile and repetitive in acute FPIES, and intermittent in chronic FPIES (9). In acute FPIES, repetitive vomiting usually occurs within 1–4 h after food ingestion, followed by diarrhea within 2–10 h, with a mean onset of approximately 5 h (10). Patients with acute FPIES often appear severely ill and may exhibit pallor, hypotonia, hypotension or shock, and/or hypothermia; however, these symptoms typically resolve within hours after feeding. In chronic FPIES, patients may present with failure to thrive, poor weight gain, weight loss, anemia, hypoproteinemia, and hypoalbuminemia. Symptom resolution often requires prolonged food avoidance, ranging from days to weeks (11). In a retrospective review of 203 patients with FPIES, 180 presented with acute disease, 8 with chronic disease, and 15 with both acute and chronic forms (12). FPIES triggered by cow's milk or soy resolves in most patients by approximately three years of age, whereas FPIES induced by solid foods often follows a more prolonged course (13). Overall, the prognosis of FPIES is favorable; a US study reported that 28% of children outgrew their FPIES trigger by 2 years, 53% by 3 years, 65% by 4 years, and 72% by 5 years (14).

FPIES is driven by non-IgE-mediated food hypersensitivity, although its precise pathophysiological mechanisms remain unclear. It is hypothesized that ingestion of food allergens triggers a *T* cell-mediated local inflammatory response, resulting in increased intestinal permeability and fluid shifts (15). Mass cytometry profiling of whole blood in children with FPIES has demonstrated activation of both innate immune cells—including monocytes, neutrophils, natural killer cells, and eosinophils—as well as T lymphocytes following food challenge (16).

Viral infections may complicate diagnosis by mimicking or coinciding with FPIES. Infections may also amplify or unmask FPIES by altering gut permeability, immune activation, or intestinal microbiota. Marzec et al. described a case of an infant with concurrent COVID-19 infection and food protein–induced allergic proctocolitis (FPIAP) (17). It has been proposed that infections may act as a priming factor for the intestinal immune system, thereby increasing the likelihood of exaggerated immune responses to dietary proteins. To date, there are no published reports clearly describing FPIES following or precipitated by a viral infection. Viral illnesses, including COVID-19, can cause transient intestinal inflammation, altered epithelial permeability, and dysregulated mucosal immunity—all of which may theoretically predispose susceptible individuals to abnormal immune responses to dietary proteins. In our case, the onset of FPIES occurred shortly after a documented COVID-19 infection in an infant who had previously tolerated cow's milk formula. Although this temporal association is intriguing, we acknowledge that it does not establish a causal relationship. Therefore, rather than suggesting that COVID-19 directly triggered FPIES, this case may represent an unmasking or cofactor role of viral infection in a genetically and immunologically susceptible infant. The initial presentation during COVID-19 infection may have mimicked viral gastroenteritis; however, the subsequent oral food challenge reproduced the classic FPIES phenotype under controlled conditions, confirming the diagnosis. This observation raises a hypothesis that post-viral immune dysregulation may contribute to the initiation of non-IgE-mediated food allergies, warranting further investigation in larger studies.

## Strengths

The patient's diagnosis was confirmed through an open oral food challenge, which elicited classic FPIES symptoms and showed rapid resolution upon elimination of the offending food. This case was reported and evaluated by experienced allergists with a clear understanding of the disease and its progression.

## Limitations

The patient's young age at initial presentation poses a limitation, making it challenging to establish a definitive causal relationship between COVID-19 infection and the onset of FPIES.

## Conclusion

Current evidence regarding the role of infections in FPIES is limited and largely anecdotal. Although infections are not established causes, the temporal association observed in this case suggests that viral infections may serve as cofactors or immune-modulating events in susceptible infants. Further studies are warranted to investigate the potential contribution of viral infections to the development or exacerbation of FPIES.

## Data Availability

The original contributions presented in the study are included in the article/Supplementary Material, further inquiries can be directed to the corresponding author.
